# Selected psychiatric problems among college students in two Arab countries: comparison with the USA

**DOI:** 10.1186/s12888-018-1718-7

**Published:** 2018-05-24

**Authors:** Ziad Kronfol, Batoul Khalifa, Brigitte Khoury, Omar Omar, Sariah Daouk, J. P. deWitt, Nourehan ElAzab, Daniel Eisenberg

**Affiliations:** 1 Weill Cornell Medicine-Qatar, Doha, Qatar; 2 Qatar University, Doha, Qatar; 3 American University of Beirut, Beirut, Lebanon; 4 University of Michigan, Ann Arbor, USA; 5 Weill Cornell Medicine, 445 E 69th St. Suite 432, New York, NY 10021 USA

**Keywords:** College students, Arab countries, Depression, Anxiety, Eating disorders, Cultural psychiatry

## Abstract

**Background:**

Psychiatric problems among college students on USA campuses are common. Little is known about similar problems in developing countries, particularly the Arab region. The goal of this study was to assess the frequency of selected psychiatric problems among college students in two Arab countries: Qatar and Lebanon, and to compare them to the USA.

**Methods:**

The Healthy Minds Study, an online confidential survey of common psychiatric symptoms designed for college campuses was used. We used the Patient Health Questionnaire-9 (PHQ-9) to screen for major depression, the Generalized Anxiety Disorder-7 (GAD-7) to screen for generalized anxiety and the SCOFF questionnaire to screen for eating disorders. Comparisons were made using ANOVA, Chi-Square tests and logistic regressions.

**Results:**

A total of 1841 students participated in the study. The rates of depression (PHQ-9 ≥ 12), generalized anxiety (GAD-7 ≥ 10) and eating disorders (SCOFF≥3) at the combined Arab universities were 34.6, 36.1 and 20.4% respectively. The corresponding rates in the USA were: 12.8, 15.9 and 6.8% (*p* < 0.001 for all measures). The impact of psychiatric problems on functioning in general and academic performance in particular was more severe in the Arab countries compared to the USA (*p* < 0.001). Independent predictors of psychiatric problems in general included location, female gender, financial difficulties and poor grades. Being religious had a protective association with mental health.

**Conclusion:**

The rates of depression, anxiety and eating disorders were significantly higher among college students in Qatar and Lebanon compared to the USA. Additional research is needed to determine whether these results reflect methodological limitations or true differences in psychopathology across these populations. If replicated, the results indicate that the psychiatric problems on college campuses in the USA are a microcosm of a global problem that needs global solutions.

## Background

The college experience is a highly desirable experience worldwide and is often associated with independence, creativity, achievements and youth. It is often seen as a rite of passage from an often tumultuous adolescence to a more stable young adulthood. However the college experience can also be very challenging to many students who are living away from home for the first time and are often facing academic pressure, financial difficulties, problems in relationships and career choices. In the USA, three quarters of all lifetime cases of mental illness begin by age 24 [1]. It is therefore not surprising that surveys of mental health problems among college students in the USA indicate a high rate of depression, anxiety and other psychiatric disorders [2]. There is also a high rate of suicidal ideation and occasional reports of completed suicide, which has led many colleges and universities across the USA to take preventive measures and increase access to counseling [2]. The success of such measures in reducing psychological morbidity and preventing suicide has not yet been firmly established.

While mental health problems among college students in developed countries such as the USA and Europe are well recognized and efforts are underway to deal with them, very little information is available from developing countries and particularly Arab countries such as Qatar, which boasts one of the highest per capita income in the world and where about half of the population is under the age of 25 [3].

The topic of college students’ mental health is not adequately covered in the Arabic literature. Substance use among college students was the topic of several reports, mostly from Lebanon [4, 5]. Anxiety symptoms among university students in the United Arab Emirates UAE were assessed using the Arabic Version of the Beck Anxiety Inventory [6]. Female students in general scored higher than their male counterparts. In a cross-national study of anxiety symptoms among college students in Beirut, Lebanon and Calgary, Canada, Lebanese students obtained higher scores than their Canadian counterparts [7]. Using the Zagazig Depression Scale (ZDS), Ibrahim and colleagues surveyed undergraduate college students at Assiut University in Egypt for depressive symptoms. They found that 37% of the students had symptoms scored above the threshold for moderate depression [8]. They also found an association between depression and low socioeconomic indices such as father’s occupation, family income and number of persons per room. The survey was done face-to-face while confidentiality of the response was assured. The same group of researchers conducted a similar survey in 6 different universities in the UK, using online methodology. They found practically the same results, pointing to the strength of the association between low socioeconomic status and depressive symptomatology in different regions and cultures [9]. To our knowledge, no comprehensive assessment of various aspects of college students’ mental health using validated instruments and modern online secure survey techniques across countries has ever been done in the Arab world. This research project is designed in part to fill this gap. We conducted a confidential online survey of selected psychiatric disorders, lifestyles, attitudes towards mental illness, and mental service utilization among college students at three universities in two Arab countries, and compared the results to a college campus in the USA.

## Methods

### Sample and data collection

The study included four universities: Education City (EC) and Qatar University (QU) in Doha, Qatar, the American University of Beirut (AUB) in Lebanon, and the University of Michigan (UM) in Ann Arbor, Michigan, USA. EC [10] is a modern hub for branches in Doha for several major universities in the USA and Europe. EC at present includes branches from Weill Cornell Medical College (medicine), Carnegie Mellon University (business and computers), Georgetown University (foreign affairs), Northwestern University (communication, journalism), Virginia Commonwealth University (art history and design), Qatar Faculty of Islamic Studies (religion), Hautes Etudes Commerciales (business) and University College London (archeology). Together EC has about 1500 students, many of them foreigners coming to study in Qatar. They come mostly from Arab or other Middle Eastern countries, Eastern European countries, or the Indian subcontinent. They also speak a variety of languages. Teaching at EC is in English. Men and women attend the same classes. QU [11], on the other hand, is a more traditional campus. Teaching is mostly in Arabic but most students speak English as well. Classes are often segregated, with men and women studying in separate classrooms. The student population is just over 17,000, the majority of whom are Qatari. AUB [12] is a private, secular and independent university. Originally founded in 1866 by Quaker missionaries as the Syrian Protestant College, it presently has about 8000 students about half of whom are Lebanese while the rest come from various, mostly Arab or Middle Eastern countries. AUB is chartered in the USA in the state of New York. The rationale for the choice of these universities is that QU represents a traditional university in a conservative Islamic country (Qatar), EC represents a more western-style, more liberal model of education in the same traditional country, while AUB offers the same model of western education in a more open and liberal Arab society (Lebanon). UM [13] on the other hand is a large, public American University located in the American Midwest. In 2017 UM celebrated its 200th anniversary.

Students were selected by simple randomization at QU, AUB and UM. The recruited sample was 6000 at QU, 3000 at AUB, and 4000 at UM. Because of the smaller population at EC, we tried to recruit all students enrolled at this site. Carnegie Mellon University-Qatar declined participation. All other universities participated, resulting in a sample size of 1200 students. The surveys were run in parallel during the spring of 2015. The selected students were sent emails inviting them to participate. All participants indicated their informed consent at the beginning of the survey. The study was approved by the institutional review board of each of the four sites.

### Sample weights

To address potential nonresponse bias, we constructed sample weights for each site’s sample, based on a small number of demographic characteristics for which the population distribution was known. For the samples at the Arab universities, these weights adjusted for the lower participation rates of men compared to women, by weighting participants by the inverse of the overall participation rate by gender. For the sample at UM, we had additional demographic information, and constructed sample weights based on gender, academic level, and grade point average. In our experience surveying university student populations, gender is the strongest predictor of survey response and therefore our estimates of the US sample do not change much if we use only gender rather than the larger set of demographics used.

### Key measures

We used the Patient Health Questionnaire-9 (PHQ-9) to measure symptoms of depression over the last 2 weeks period. This self-report scale, which is based on the DSM-IV, has been used to measure both the severity of depressive symptoms as well as the diagnosis of major depression [14]. The scale has been shown to have good sensitivity and specificity [15]. It has been used in different settings and has been translated into different languages including Arabic and validated in different cultures including Saudi Arabia [16] and Lebanon [17]. Scores on the PHQ-9 are usually interpreted as follows: score 0–4: no depression; score 5–9: mild depression; score 10–14: moderate depression and score ≥ 15: severe depression. We have used the PHQ-9 as a screen for the diagnosis for major depression. Although different cutoff points have been suggested, we used the cutoff of 12 as the best cutoff both for sensitivity and specificity in an Arab country [18].

We used the Generalized Anxiety Disorder-7 (GAD-7) to measure symptoms of anxiety over the last 2 weeks. Like the PHQ-9, the GAD-7 is a self-report questionnaire based on DSM-IV criteria of generalized anxiety disorder [19]. This scale was also shown to have good sensitivity but specificity has been variable [20]. This scale has also been used in different settings and in different countries including Lebanon [17]. Scores on the GAD-7 were interpreted as follows: score 0–4: no anxiety; score 5–9: mild anxiety; score 10–14: moderate anxiety and score ≥ 15: severe anxiety. As with the PHQ-9, we also used the GAD-7 for the diagnosis of generalized anxiety disorder. We also chose a cutoff of 10 for this diagnosis, in line with similar studies conducted in Arab countries [17].

We used the SCOFF questionnaire [21] as a screening tool for eating disorders at the time of the study. The SCOFF is comprised of 5 leading questions covering different aspects of an eating disorder. A “yes” answer to 3 questions or more is highly suggestive of the presence of anorexia nervosa or bulimia. The SCOFF has been used in a variety of settings and in different countries, and has been validated for use in Lebanon [22].

We also included other demographic and personal measures such as living arrangements, financial situation and religiosity. Many of these measures are self-explanatory (e.g. living in dorms, off-campus single or sharing or off-campus with parent, spouse or other relative), Some measures require a certain amount of subjectivity (e.g. current financial situation: “it’s a financial struggle”; “tight but doing fine” or “finances aren’t a problem” and religiosity: “very religious”; “fairly religious”; “not too religious” or “not religious at all”). For measurement of functional impairments caused by mental health problems we used two items. First, we included an item that usually follows the PHQ-9: “If you checked off any problems, how difficult have these problems made it for you to do your work, take care of things at home, or get along with other people?” Second, we included a question about impairment in academic performance: “In the past 4 weeks, how many days have you felt that emotional or mental difficulties have hurt your academic performance?” These measures complement the PHQ-9 and the GAD-7 screens for clinical depression and anxiety and have frequently been used in college surveys screening for mental health problems among students [2].

### Statistical analysis

Chi-squared tests were used to determine the differences between schools (Arab and USA) for binary outcomes. Differences between schools for ordinal outcomes were calculated by using chi-squared test for trend. Multivariable logistic regression with random intercept models was used to test for independent associations between outcomes (depression/ anxiety/ eating disorders) and variables of interest taking account of the clustering effect of schools by using Stata 13 command *xtlogit*. Main effects and interactions between correlates and schools were examined for each outcome. All analyses were done using Stata 13 (Stata Corp, College Station, Texas, USA) and using the svy commands to account for the sampling weights as discussed above.

## Results

### Basic sample characteristics

Fourteen thousand two-hundred students were recruited during the spring of 2015. Participation rates were as follows: EC 351/1200 (29%); QU: 434/6000 (7.2%); AUB: 167/3000 (5.6%) and UM: 889/4000 (22.2%) for a total participation rate of 1841/14,200 (13.0%). Participants’ characteristics are shown in Table 1. As expected, there were major differences in the basic profile of the students at the different sites. This was our intention, as we wanted to compare campuses with different cultural, ethnic, and socioeconomic characteristics.

**Table 1:** Participants characteristics, in weighted percentages (Standard error)

Characteristic	Education City (*n* = 351)	Qatar University (*n* = 434)	American University of Beirut (*n* = 167)	University of Michigan (*n* = 889)	*P*-value
Age in years					
18–20	36.5 (3.5)	33.8 (2.3)	64.0 (3.9)	35.7 (1.8)	< 0.001
21–25	42.1 (3.5)	46.7 (2.5)	30.8 (3.7)	42.3 (1.8)	
> =26	21.4 (2.5)	19.5 (2.0)	5.3 (1.9)	22.0 (1.4)	
Gender					
Female	58.3 (3.5)	71.9 (2.3)	50.3 (4.1)	47.4 (1.8)	< 0.001
Nationality					
Nationals of the host country	47.3 (3.6)	71.5 (2.3)	83.5 (3.0)	86.6 (1.2)	< 0.001
Program					
Undergraduate	76.7 (2.6)	83.6 (2.0)	93.7 (1.8)	65.2 (1.7)	< 0.001
Living arrangements					
On campus Dorms	25.7 (3.2)	1.2 (0.6)	16.8 (3.1)	21.8 (1.6)	< 0.001
Off-campus, single or sharing	7.7 (1.5)	3.8 (1.0)	18.0 (3.2)	71.2 (1.7)	
Off-campus, parents, spouse or other relatives	66.7 (3.4)	95.1 (1.2)	65.3 (3.9)	7.0 (0.9)	
In a relationship					
Yes	31.7 (3.4)	29.7 (2.5)	23.7 (3.5)	46.0 (1.8)	0.0259
Current financial situation					
It’s a financial struggle	10.6 (2.3)	3.6 (1.0)	9.4 (2.4)	10.2 (1.1)	< 0.001
Tight but doing fine	46.5 (3.8)	42.3 (2.6)	54.2 (4.1)	50.7 (1.8)	
Finances aren’t a problem	42.9 (3.7)	54.1 (2.6)	36.4 (3.9)	39.2 (1.8)	
GPA					
A+ to A-	55.8 (3.9)	22.2 (2.2)	33.5 (3.9)	59.2 (1.9)	< 0.001
B+ to B-	39.6 (3.9)	50.6 (2.7)	56.8 (4.1)	37.2 (1.9)	
C+ and below	4.5 (1.6)	27.2 (2.4)	9.7 (2.5)	3.7 (0.8)	
Religiosity					
Fairly/Very religious	87.0 (2.3)	94.7 (1.2)	43.7 (4.0)	32.5 (1.7)	< 0.001
Not too religious/Not religious at all	13.0 (2.3)	5.3 (1.2)	56.3 (4.0)	67.5 (1.7)	

The students in the Arab universities tended to be slightly younger, likely because of the higher proportion of undergraduates, particularly at AUB. Interestingly, there was also a higher representation by women. The universities in Qatar had fewer nationals compared to both AUB and UM. More students in the Arab universities lived with their parents or relatives and fewer at the dorms. Also, more students at UM reported being in a relationship. Financially, the students in Qatar reported being better off than students both in Lebanon and the USA. Academically, the students both in EC and at UM reported having somewhat better grades. Also, the students in Qatar identified as being more religious than their counterparts in Lebanon or the USA. Many of these differences were expected as the sites were chosen to reflect different cultures and sensitivities.

### Overall prevalence of depression, anxiety and eating disorders

The prevalence rates for major depression (PHQ-9 ≥ 12), generalized anxiety (GAD≥10) and eating disorders (SCOFF≥3) in EC, QU, AUB and UM are shown in Table 2. The rates of major depression, generalized anxiety and eating disorder were very similar in EC, QU and AUB compared to UM. The corresponding rate (SE) for major depression in EC was 40.5% (4.1), in QU was 32.0% (2.7), and at AUB was 38.6% (4.1). The rate at UM was much smaller at 12.8%.(1.3)), a difference which is statistically significant (*p* < 0.001). The corresponding rate (SE) for generalized anxiety disorder in EC was 38.3%, (3.6), in QU was 34.2%,(2.8) and at AUB was 39.4% (4.2), significantly higher than at UM where the rate was 15.9% (1.4), a difference which was statistically significant (*p* < 0.001). Similar trends were also noted for eating disorders, with corresponding rates (SE) of 18.8% (4.2), 21.4% (2.4) and 18.6% (3.2) at EC, QU and AUB respectively, rates significantly higher than at UM, with a rate of only 6.8% (0.9). These differences were also statistically significant (*p* < 0.001). Questions regarding functional impairment caused by depression and academic performance being affected by mental health problems are also shown in Table 2. The functional impairment was higher in EC, QU and AUB than at UM (*p* < 0.001). Similarly, the students at EC, QU and AUB report their academic performance has been more seriously affected than students at UM (*p* < 0.001).

**Table 2:** Prevalence of psychiatric problems and consequences in Qatar, Lebanon and the USA, in weighted percentage (Standard error)

	Education City (*n* = 351)	Qatar University (*n* = 434)	American University of Beirut (*n* = 167)	Arab Universities combined (*n* = 952)	University of Michigan (*n* = 889)	*p*-value
Major Depression (PHQ-9)						
No (0–11)	59.5 (4.1)	68.0 (2.7)	61.4 (4.1)	65.4 (2.1)	87.2 (1.3)	< 0.001
Yes (≥12)	40.5 (4.1)	32.0 (2.7)	38.6 (4.1)	34.6 (2.1)	12.8 (1.3)	
Generalized Anxiety (GAD-7)						
No (0–9)	61.7 (4.2)	65.8 (2.8)	60.6 (4.2)	63.9 (2.2)	84.1 (1.4)	< 0.001
Yes (≥10)	38.3 (4.2)	34.2 (2.8)	39.4 (4.2)	36.1 (2.2)	15.9 (1.4)	
Eating disorder (SCOFF)						
No (0–2)	81.2 (4.2)	78.6 (2.4)	81.4 (3.2)	79.6 (1.8)	93.2 (0.9)	< 0.001
Yes (≥3)	18.8 (4.2)	21.4 (2.4)	18.6 (3.2)	20.4 (1.8)	6.8 (0.9)	
Function impairments caused by depression						
Not at all difficult	20.6 (3.1)	26.1 (2.5)	24.1 (3.7)	25.2 (1.9)	40.9 (1.9)	< 0.001
Somewhat difficult	51.7 (4.2)	59.0 (2.8)	54.6 (4.2)	57.3 (2.2)	48.6 (2.0)	
Very difficult	18.4 (3.3)	11.3 (1.8)	15.0 (3.1)	12.9 (1.5)	9.2 (1.2)	
Extremely difficult	9.3 (2.7)	3.5 (1.0)	6.2 (1.9)	4.7 (0.9)	1.3 (0.4)	
MH affected academic performance						
0 days	25.4 (3.3)	21.5 (2.5)	21.4 (3.5)	21.7 (1.9)	45.6 (1.9)	< 0.001
1–2 days	23.0 (3.6)	32.4 (2.8)	25.9 (3.8)	29.7 (2.1)	28.2 (1.7)	
3–5 days	19.0 (3.2)	24.4 (2.6)	23.2 (3.6)	23.7 (2.0)	16.1 (1.4)	
≥ 6 days	32.6 (4.1)	21.6 (2.5)	29.5 (3.8)	24.8 (2.0)	10.2 (1.1)	

**Fig. 1:**
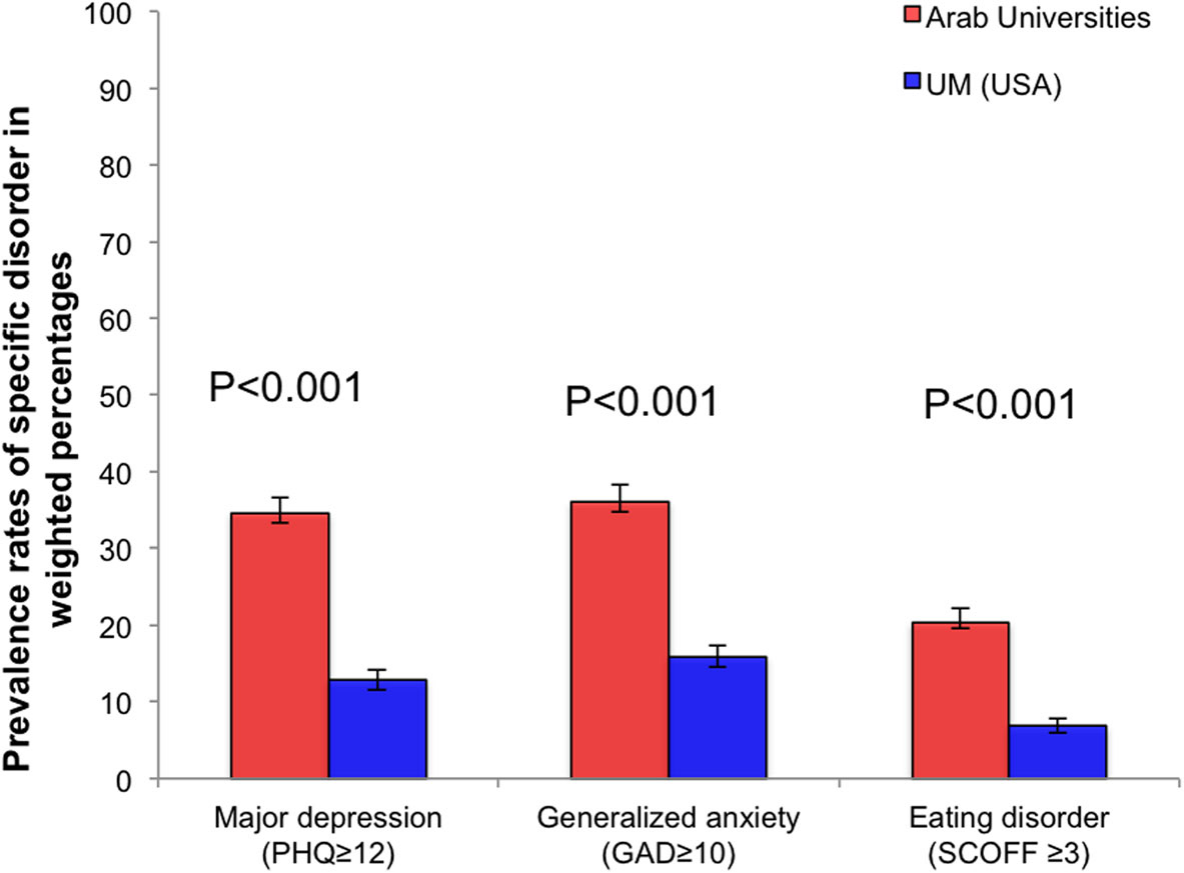
Prevalence rates (in weighted percentages +/− SE) of major depression, generalized anxiety and eating disorders among college students in Arab countries and the USA

Because of the similarities in the rates of depression, anxiety and eating disorders in the three Arab sites (none of the differences were statistically significant), we combined the three Arab universities together and compared them with the one University in the USA. The results are shown graphically in Fig. 1. These results show that the rates of major depression of 34.6% (2.1), generalized anxiety 36.1% (2.2) and eating disorder 20.4% (1.8) on college campuses in Arab countries are significantly higher (*p* < 0.001) than corresponding rates in the USA of 12.8% (1.3), 15.9% (1.4) and 6.8% (0.9) respectively.

### Independent correlates of psychiatric problems on university campuses

We then looked at independent predictors of major depression, generalized anxiety and eating disorder in our entire sample (both Arab campuses and the USA) (Table 3). Significant predictors of major depression included location in the two Arab countries (OR: 4.82; CI: 4.01, 5.79), being a woman (OR: 1.88; CI: 1.72, 2.05), having financial difficulties (OR: 7.63; CI: 3.50, 16.65) and having low grades (OR: 2.79; CI: 0.74, 10.55). Being in a relationship was a protective factor (OR: 0.90; CI: 0.83, 0.97). Being fairly or very religious was another independent protective factor (OR: 0.57; CI: 0.38, 0.86).

**Table 3:** Adjusted Odds Ratios (OR) and 95% Confidence Intervals (CIs) for predictors of psychiatric problems

	Depression (PHQ ≥ 12) (*n* = 1312)	Anxiety (GAD ≥ 10) (*n* = 1266)	Eating disorders (*n* = 571)
	OR (95% CI)	OR (95% CI)	OR (95% CI)
UM	1.00	1.00	1.00
Arab	4.82 (4.01, 5.79)^b^	2.72 (1.52, 4.86)^b^	1.98 (1.16, 3.40)^a^
Age 18–20	1.00	1.00	1.00
Age 21–25	1.13 (0.77, 1.66)	0.75 (0.36, 1.54)	1.04 (0.60, 1.79)
Age 26–30	1.23 (0.76, 1.97)	1.46 (1.14, 1.86)^b^	2.19 (0.63, 7.62)
Female	1.88 (1.72, 2.05)^b^	2.40 (2.17, 2.67)^b^	2.52 (1.40, 4.51)^b^
Dorms	1.00	1.00	1.00
Off-campus, single or sharing	1.33 (0.61, 2.89)	1.18 (0.90, 1.55)	0.82 (0.46, 1.46)
Off-campus, parent, relative or spouse	1.07 (0.51, 2.24)	1.86 (1.03, 3.36)^a^	1.69 (1.00, 2.85)
Graduate	0.78 (0.57, 1.08)	0.52 (0.35, 0.78)^b^	0.31 (0.10, 0.97)^a^
Finances aren’t a problem	1.00	1.00	1.00
Tight but doing fine	1.57 (1.16, 2.13)^b^	1.86 (1.79, 1.94)^b^	1.35 (0.79, 2.30)
It’s a financial struggle	7.63 (3.50, 16.65)^b^	12.10 (7.45, 19.64)^b^	2.83 (1.11, 7.24)^a^
In a relationship	0.90 (0.83, 0.97)^b^	0.93 (0.63, 1.38)	1.31 (0.80, 2.13)
GPA			
A+ to A-	1.00	1.00	1.00
B+ to B-	1.60 (1.08, 2.38)^a^	1.35 (1.11, 1.64)^b^	0.78 (0.38, 1.60)
C+ and below	2.79 (0.74, 10.55)^b^	1.51 (0.74, 3.09)	0.94 (0.49, 1.79)
Not religious or not religious at all	1.00	1.00	1.00
Fairly or very religious	0.57 (0.38, 0.86)^b^	0.46 (0.31, 0.71)^b^	0.89 (0.67, 1.18)
National of country	1.00 (0.78, 1.29)	0.98 (0.79, 1.21)	1.19 (0.67, 2.13)

^a^significant at 5% level

^b^significant at 1% level

Significant predictors for generalized anxiety again included location in the two Arab countries (OR: 1.98; CI: 1.16, 3.40), being a woman (OR: 2.40; CI: 2.17, 2.67) and having financial difficulties (OR: 12.10; CI: 7.45, 19.64). Other predictors of generalized anxiety include older age (OR: 1.46; CI: 1.14, 1.86) and living off campus with a parent, relative or a spouse (OR: 1.86; CI: 1.03, 3.36) and being a B student (OR: 1.35; CI: 1.11, 1.64). Protective factors against anxiety include again being religious (OR: 0.46: CI: 0.31, 0.71), but also being a graduate student (OR: 0.52; CI: 0.35, 0.78).

Predictors for eating disorders again include location in the two Arab countries (OR: 1.98; CI: 1.16, 3.40), female gender (OR: 2.52; CI: 1.40, 4.51) and having financial difficulties (OR: 2.83; CI: 1.11, 7.24). One protective factor is being a graduate student (OR: 0.31; CI: 0.10, 0.97).

We also looked at interactions (data not shown) between the different variables, the outcome measures (depression, anxiety and eating disorders) and school location (Arab vs USA). We found interesting significant interactions (*p* < 0.05) between gender and school for all three outcomes, with female gender being more closely associated with psychopathology in the three Arab universities compared to the USA. There were also significant interactions between financial difficulties and both depression (*p* < 0.001) and anxiety (*p* < 0.001) but not eating disorders with respect to school location, with more than double the rates of both depression and anxiety among students who are struggling financially and living in Arab countries compared to those living in the USA. We also found significant interactions between academic performance, the various outcomes and school location, with low grades again being more strongly associated with psychopathology in the Arab countries (*p* < 0.001) compared to the USA.

## Discussion

The most notable finding from this study is the high prevalence of probable major depression (34.6%), generalized anxiety (36.1%), and eating disorders (20.4%) reported by a sample of college students in Arab countries compared to the USA, with corresponding rates of 12.8, 15.9 and 6.8% respectively. It is also important to note that these disorders seem to cause more functional impairment and are more likely to affect academic performance in Arab countries compared to the USA. These results are of particular interest when put in context: the three universities in the Arab countries are quite different in terms of history, location, educational philosophy and student population. We therefore expected the rate of psychopathology for instance in Lebanon to be somewhat halfway between the corresponding rates in Qatar and the USA. Yet the prevalence of three of the most common psychiatric disorders among the students in the two Arab countries is very similar. The prevalence of these disorders among the students in Qatar and Lebanon is 2–3 times larger than in the USA. Furthermore, the survey was done online using the same survey format at approximately the same time in all four locations, adding to the validity of the findings.

How do results from this survey compare with other surveys from Arab countries? In a recent study on the prevalence of depression and anxiety among Saudi university students using the PHQ-9 and the standard algorithm for depression, the authors report symptoms of major depression in 9.9% of the students, symptoms of other depression in 19.4%, and any depression in 24.4% of the students [23]. Again using the PHQ, they report generalized anxiety in 14.0% of the students. Part of the discrepancy with our results may be related to gender differences, as their sample consisted mostly of men who reported significantly lower rates of both major depression and generalized anxiety than women. Again using the PHQ-9 with a cut-off of 12, Al-Ghafri and colleagues estimated the prevalence of depression among medical trainees in Oman to be 11.4%. [18]. As to the prevalence of depression in Saudi primary care adult patients, the rates for depression using PHQ-9 were 49.9%, of which 31% had mild symptoms, 13.4% moderate, 4.4% moderate to severe and 1.0% severe symptoms [24]. As mentioned earlier, using the ZDS rating scale, Ibrahim and colleagues reported a depression rate of 37% among undergraduate students at Assiut University in Egypt [8].

Regarding the behavioral rating scales we used, namely the PHQ-9, the GAD-7 and the SCOFF, although they have all been validated for use in Arab countries, there is still some controversy regarding the cut-off point to be used for diagnostic purposes, particularly with the PHQ-9 for depression. In general, the lower the cutoff point, the larger the rate of a particular diagnosis. In our study, we used these instruments both for severity measurements and disease prevalence. However, because this is a comparative study, the conclusion that the rates of depression, anxiety and eating disorders are higher in Arab countries remains valid regardless of the cutoff point used. Some researchers have used the standard algorithm for interpreting the PHQ-9 [25], and accordingly diagnosed the subjects with major depression, minor depression or no depression. We examined our data along these lines and reached the same conclusion: that although the rates for specific disorders could change, these disorders are substantially more common in Arab countries compared to the USA (data not shown).

In a systematic review of depression prevalence among university students worldwide [26], the rates of depression varied between 10.3 and 84.5% with a mean weighted prevalence of 30.6%. The most common scales used were the Beck Depression Inventory BDI [27] followed by the Center for Epidemiological Studies-Depression CES-D [28] and the PHQ-9. The authors concluded that the rates of depression experienced by university students are substantially higher than those found in the general population [26]. In a recent validation study of the PHQ-9 and GAD-7 in a Lebanese psychiatric clinic, the authors conclude that both the PHQ-9 and the GAD-7 are reliable screening tools for depression and anxiety in an Arabic population, but that the diagnostic specificity of these instruments is not very robust. Furthermore, the authors believe the PHQ-9 tends to exaggerate depressive symptomatology in the Arabic population or that it may be sensitive to sub-threshold symptoms [17].

Direct comparison of psychiatric symptoms between Arab and American universities is rare but results similar to our own have been reported by Abdel-Khalek and Lester who found anxiety among Kuwaiti college students to be higher than in their American counterparts [29]. One possible explanation could be the high rate of comorbidity in the Arab sample. Co-occurrence rates (at least two occurrences) were elevated, both in the Arab countries and the USA but were more elevated among students in Arab countries. This could account, at least in part, for the inflation seen in the Arab countries. Among students with an eating disorder, for instance, 64.0% reported a co-occurring condition (depression or anxiety) in Arab countries. The corresponding rate was 38.2% at UM.

When we looked at independent risk factors that may be associated with depression, anxiety and eating disorders among college students on all campuses, several interesting findings were noted. First, location (being in the two Arab countries) was a significant predictor for all three outcomes, consistent with data presented in Table 2. Similarly, being a woman was a significant risk factor associated with all three conditions, a finding in line with most epidemiological studies regarding these conditions [23, 30]. Another major predictor of mental health problems across countries seems to be financial difficulties, which is associated with depression, anxiety and eating disorders on all campuses. This is somewhat surprising as many students, particularly in Qatar, described their current financial situation as “finances aren’t a problem”, yet for those who struggle financially there is an increased risk for depression, anxiety and eating disorder both in Arab countries and the USA. In that regard, our results seem to agree with Amr and colleagues who reported that female gender, financial and personal problems were significant predictors of depression in Saudi university students [23]. Another interesting predictor of psychopathology is the effect of academic performance [31]: low grades (C+ and below) constitute an independent risk factor for depression while being a B student increases the risk for anxiety. Perhaps the B performance keeps many students on their guards, producing more anxiety, while the C and below performance is more likely to lead to despair and depression.

There were also factors that seem to provide some protection against psychopathology. For instance, being a graduate student seems to be associated with a lower risk for both anxiety and eating disorder. This could be a sign of better adaptation to college life but more data are needed in that regard. Similarly, being in a relationship could reduce the risk for depression, as loneliness is a factor that is frequently associated with depression. Religiosity is another factor that seems to be associated with a lower risk for both depression and anxiety. This is consistent with other reports from both Arab countries [32] and the USA [33] as religiosity can be understood as providing a sense of purpose and support for students everywhere [34].

This study has a number of limitations. First, the survey participation rate was very low (under 10%) at two of the sites (AUB and QU). The rate was substantially higher at EC, however, and the prevalence estimates were similar across all three Arab universities. We expected the participation to be lower in Arab countries as the students are not used to this type of research. Our sample weights can at least correct for a limited set of demographic characteristics that predict participation, such as gender. Second, the universities we surveyed may not be representative of the regions we cover. We chose three very different universities in two very different Arab countries. The demographic characteristics were very different. Yet the prevalence of depression, anxiety and eating disorder were very similar. Obviously more countries need to be included in future studies using similar methodologies to better map-out the prevalence of these common psychiatric disorders. There is also the possibility that UM does not adequately represent all US universities, although previous studies suggest that it is near the middle of the national distribution in terms of mental health prevalence [2]. Lastly, we used measures of mental health that although validated in the different countries, were based on brief self-reported instruments that may not correlate perfectly with clinicians’ diagnoses.

## Conclusions

In summary, our work shows that psychiatric problems so common on college campuses in Western countries are not limited to those countries. In fact, these problems may be even more common and more serious in developing countries, where students often face greater financial, political and/or other psychosocial challenges and lack the services and/or resources to deal with them. We are presently looking at mental health service utilization in those countries and the reasons why the people who need them the most are not accessing them the way they should. Some answers may be local in nature and call for specific legislation in a specific country or region while others may be more general in nature and call for more global attention and intervention.

## Abbreviations

AUB: American University of Beirut
EC: Education City
GAD-7: Generalized Anxiety Disorder-7
PHQ-9: Patient Health Questionnaire-9
QU: Qatar University
UM: University of Michigan
